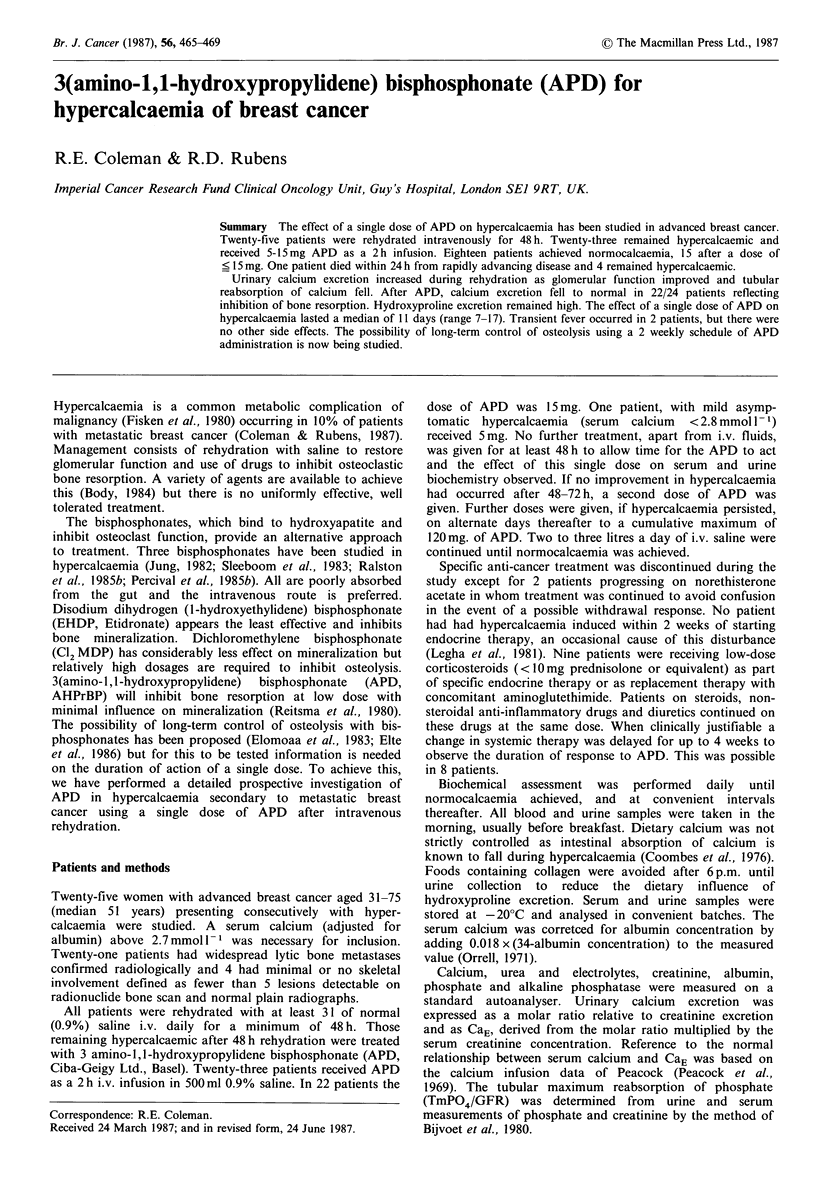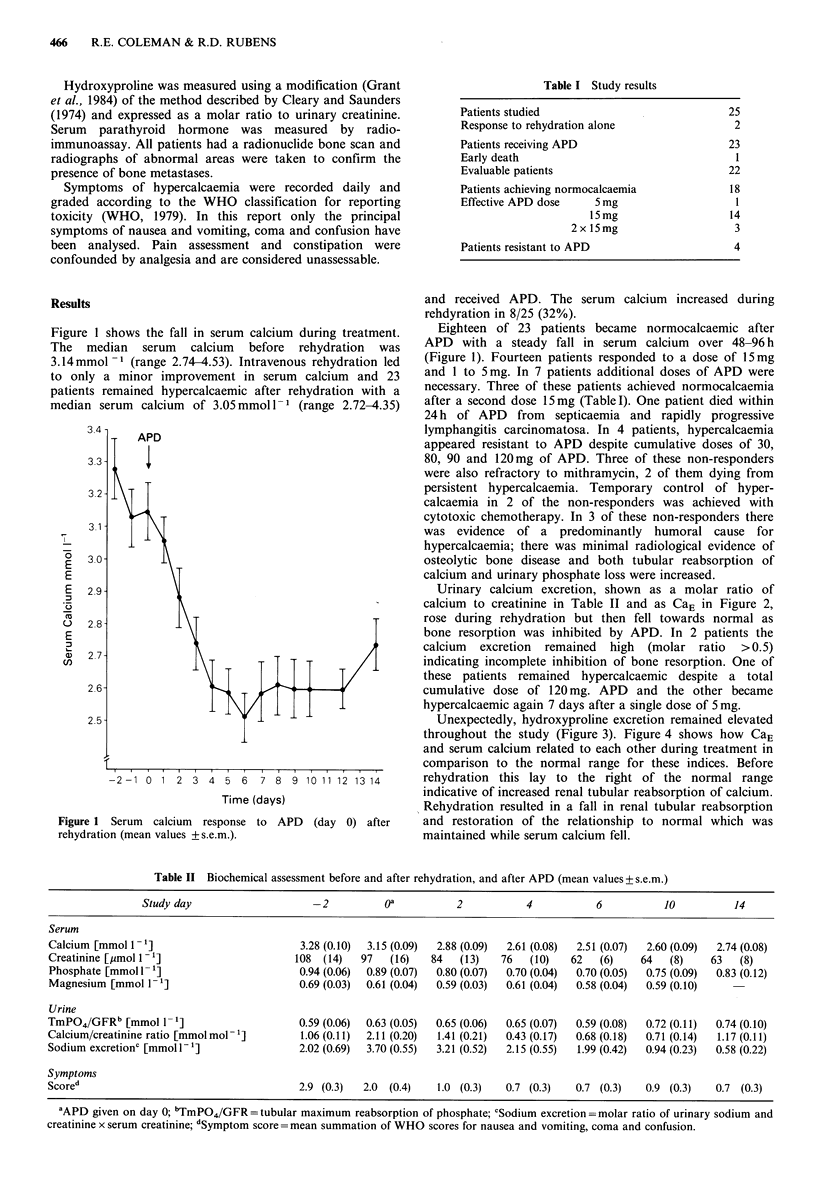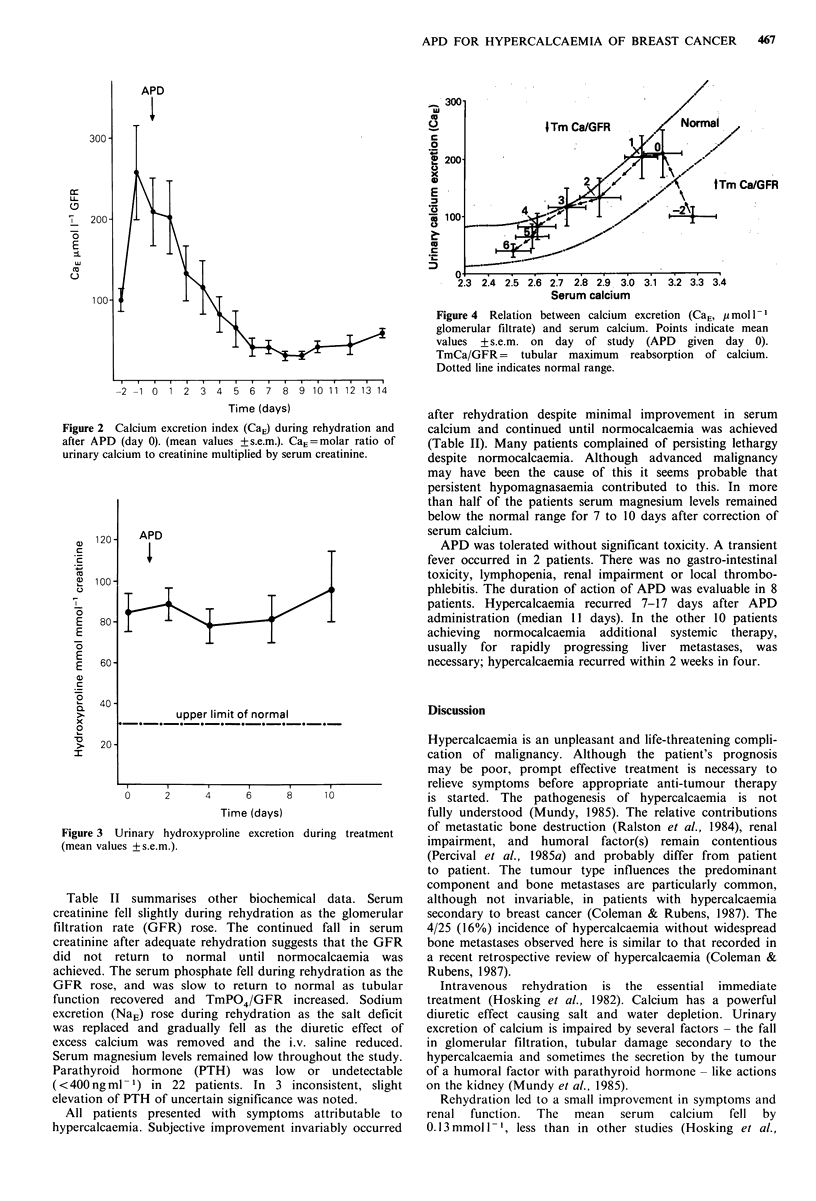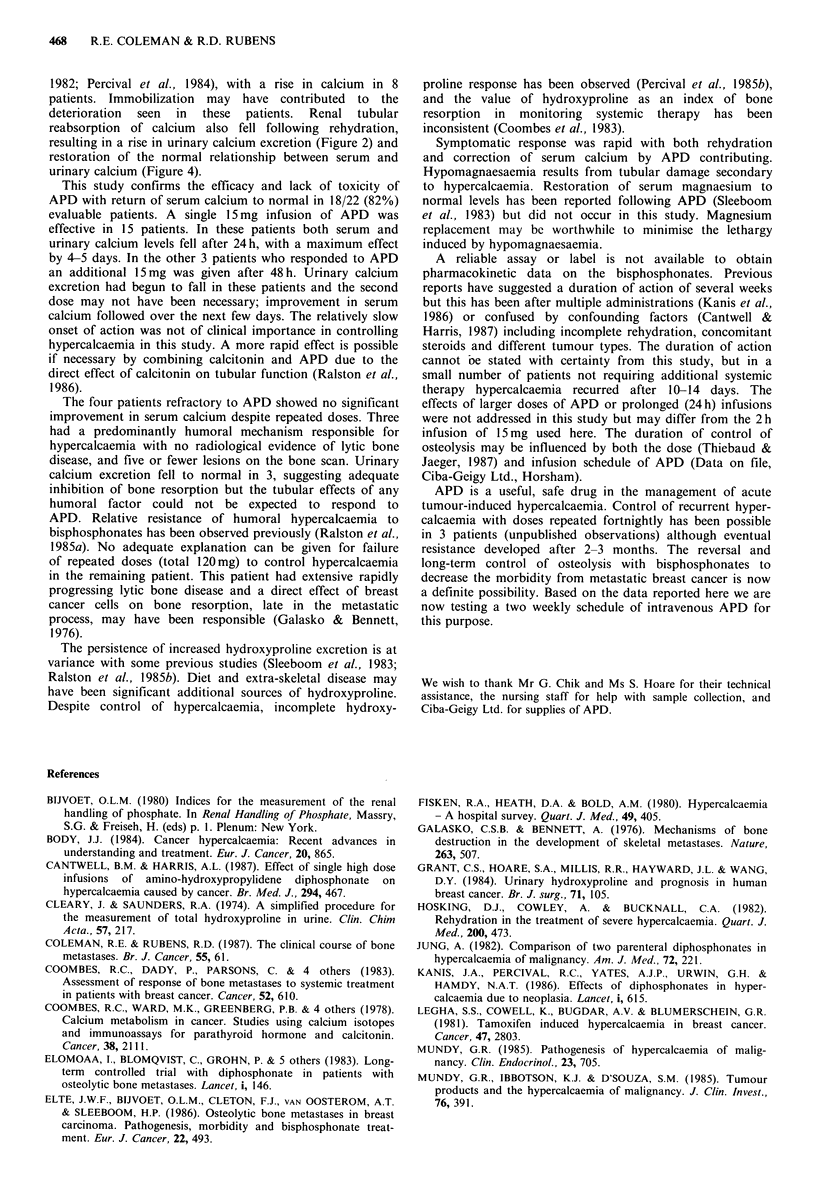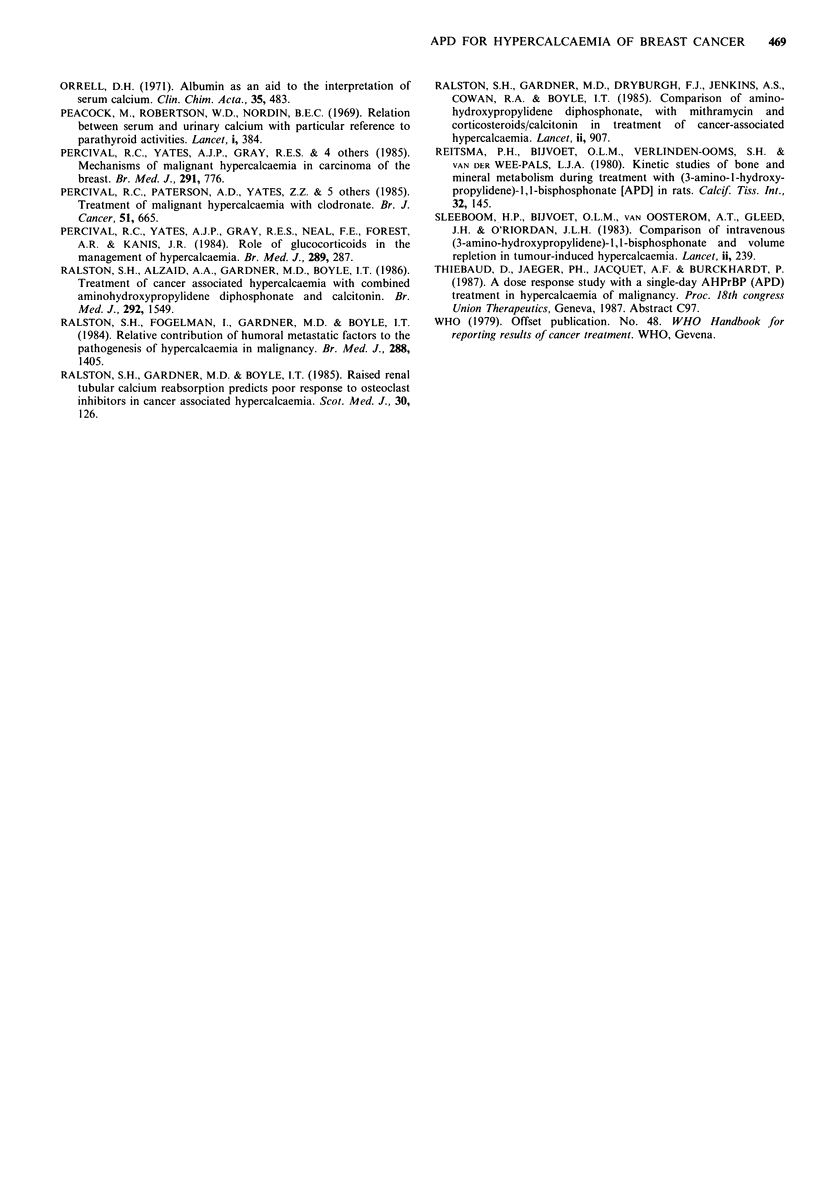# 3(Amino-1,1-hydroxypropylidene) bisphosphonate (APD) for hypercalcaemia of breast cancer.

**DOI:** 10.1038/bjc.1987.225

**Published:** 1987-10

**Authors:** R. E. Coleman, R. D. Rubens

**Affiliations:** Imperial Cancer Research Fund Clinical Oncology Unit, Guy's Hospital, London, UK.

## Abstract

The effect of a single dose of APD on hypercalcaemia has been studied in advanced breast cancer. Twenty-five patients were rehydrated intravenously for 48 h. Twenty-three remained hypercalcaemic and received 5-15 mg APD as a 2 h infusion. Eighteen patients achieved normocalcaemia, 15 after a dose of less than or equal to 15 mg. One patient died within 24 h from rapidly advancing disease and 4 remained hypercalcaemic. Urinary calcium excretion increased during rehydration as glomerular function improved and tubular reabsorption of calcium fell. After APD, calcium excretion fell to normal in 22/24 patients reflecting inhibition of bone resorption. Hydroxyproline excretion remained high. The effect of a single dose of APD on hypercalcaemia lasted a median of 11 days (range 7-17). Transient fever occurred in 2 patients, but there were no other side effects. The possibility of long-term control of osteolysis using a 2 weekly schedule of APD administration is now being studied.


*Br. J. Cancer* (1987), **56**, 465–469 © The Macmillan Press Ltd., 1987

### 3(amino-1,1-hydroxypropylidene) bisphosphonate (APD) for hypercalcaemia of breast cancer

R.E. Coleman & R.D. Rubens

*Imperial Cancer Research Fund Clinical Oncology Unit, Guy's Hospital, London SE1 9RT, UK.*

**Summary** The effect of a single dose of APD on hypercalcaemia has been studied in advanced breast cancer. Twenty-five patients were rehydrated intravenously for 48 h. Twenty-three remained hypercalcaemic and received 5-15 mg APD as a 2 h infusion. Eighteen patients achieved normocalcaemia, 15 after a dose of ≦15 mg. One patient died within 24 h from rapidly advancing disease and 4 remained hypercalcaemic.

Urinary calcium excretion increased during rehydration as glomerular function improved and tubular reabsorption of calcium fell. After APD, calcium excretion fell to normal in 22/24 patients reflecting inhibition of bone resorption. Hydroxyproline excretion remained high. The effect of a single dose of APD on hypercalcaemia lasted a median of 11 days (range 7–17). Transient fever occurred in 2 patients, but there were no other side effects. The possibility of long-term control of osteolysis using a 2 weekly schedule of APD administration is now being studied.

Hypercalcaemia is a common metabolic complication of malignancy (Fisken *et al.*, 1980) occurring in 10% of patients with metastatic breast cancer (Coleman & Rubens, 1987). Management consists of rehydration with saline to restore glomerular function and use of drugs to inhibit osteoclastic bone resorption. A variety of agents are available to achieve this (Body, 1984) but there is no uniformly effective, well tolerated treatment.

The bisphosphonates, which bind to hydroxyapatite and inhibit osteoclast function, provide an alternative approach to treatment. Three bisphosphonates have been studied in hypercalcaemia (Jung, 1982; Sleeboom *et al.*, 1983; Ralston *et al.*, 1985*b*; Percival *et al.*, 1985*b*). All are poorly absorbed from the gut and the intravenous route is preferred. Disodium dihydrogen (1-hydroxyethylidene) bisphosphonate (EHDP, Etidronate) appears the least effective and inhibits bone mineralization. Dichloromethylene bisphosphonate ($Cl_2$ MDP) has considerably less effect on mineralization but relatively high dosages are required to inhibit osteolysis. 3(amino-1,1-hydroxypropylidene) bisphosphonate (APD, AHPrBP) will inhibit bone resorption at low dose with minimal influence on mineralization (Reitsma *et al.*, 1980). The possibility of long-term control of osteolysis with bisphosphonates has been proposed (Elomoaa *et al.*, 1983; Elte *et al.*, 1986) but for this to be tested information is needed on the duration of action of a single dose. To achieve this, we have performed a detailed prospective investigation of APD in hypercalcaemia secondary to metastatic breast cancer using a single dose of APD after intravenous rehydration.

### Patients and methods

Twenty-five women with advanced breast cancer aged 31–75 (median 51 years) presenting consecutively with hypercalcaemia were studied. A serum calcium (adjusted for albumin) above 2.7 $mmol\,l^{-1}$ was necessary for inclusion. Twenty-one patients had widespread lytic bone metastases confirmed radiologically and 4 had minimal or no skeletal involvement defined as fewer than 5 lesions detectable on radionuclide bone scan and normal plain radiographs.

All patients were rehydrated with at least 3 l of normal (0.9%) saline i.v. daily for a minimum of 48 h. Those remaining hypercalcaemic after 48 h rehydration were treated with 3 amino-1,1-hydroxypropylidene bisphosphonate (APD, Ciba-Geigy Ltd., Basel). Twenty-three patients received APD as a 2 h i.v. infusion in 500 ml 0.9% saline. In 22 patients the dose of APD was 15 mg. One patient, with mild asymptomatic hypercalcaemia (serum calcium <2.8 $mmol\,l^{-1}$) received 5 mg. No further treatment, apart from i.v. fluids, was given for at least 48 h to allow time for the APD to act and the effect of this single dose on serum and urine biochemistry observed. If no improvement in hypercalcaemia had occurred after 48–72 h, a second dose of APD was given. Further doses were given, if hypercalcaemia persisted, on alternate days thereafter to a cumulative maximum of 120 mg. of APD. Two to three litres a day of i.v. saline were continued until normocalcaemia was achieved.

Specific anti-cancer treatment was discontinued during the study except for 2 patients progressing on norethisterone acetate in whom treatment was continued to avoid confusion in the event of a possible withdrawal response. No patient had had hypercalcaemia induced within 2 weeks of starting endocrine therapy, an occasional cause of this disturbance (Legha *et al.*, 1981). Nine patients were receiving low-dose corticosteroids (<10 mg prednisolone or equivalent) as part of specific endocrine therapy or as replacement therapy with concomitant aminoglutethimide. Patients on steroids, non-steroidal anti-inflammatory drugs and diuretics continued on these drugs at the same dose. When clinically justifiable a change in systemic therapy was delayed for up to 4 weeks to observe the duration of response to APD. This was possible in 8 patients.

Biochemical assessment was performed daily until normocalcaemia achieved, and at convenient intervals thereafter. All blood and urine samples were taken in the morning, usually before breakfast. Dietary calcium was not strictly controlled as intestinal absorption of calcium is known to fall during hypercalcaemia (Coombes *et al.*, 1976). Foods containing collagen were avoided after 6 p.m. until urine collection to reduce the dietary influence of hydroxyproline excretion. Serum and urine samples were stored at −20°C and analysed in convenient batches. The serum calcium was corretced for albumin concentration by adding 0.018 × (34-albumin concentration) to the measured value (Orrell, 1971).

Calcium, urea and electrolytes, creatinine, albumin, phosphate and alkaline phosphatase were measured on a standard autoanalyser. Urinary calcium excretion was expressed as a molar ratio relative to creatinine excretion and as $Ca_E$, derived from the molar ratio multiplied by the serum creatinine concentration. Reference to the normal relationship between serum calcium and $Ca_E$ was based on the calcium infusion data of Peacock (Peacock *et al.*, 1969). The tubular maximum reabsorption of phosphate ($TmPO_4$/GFR) was determined from urine and serum measurements of phosphate and creatinine by the method of Bijvoet *et al.*, 1980.

Correspondence: R.E. Coleman.
Received 24 March 1987; and in revised form, 24 June 1987.

Hydroxyproline was measured using a modification (Grant *et al.*, 1984) of the method described by Cleary and Saunders (1974) and expressed as a molar ratio to urinary creatinine. Serum parathyroid hormone was measured by radioimmunoassay. All patients had a radionuclide bone scan and radiographs of abnormal areas were taken to confirm the presence of bone metastases.

Symptoms of hypercalcaemia were recorded daily and graded according to the WHO classification for reporting toxicity (WHO, 1979). In this report only the principal symptoms of nausea and vomiting, coma and confusion have been analysed. Pain assessment and constipation were confounded by analgesia and are considered unassessable.

**Table I** Study results

| | | |
|---|---|---|
| Patients studied | | 25 |
| Response to rehydration alone | | 2 |
| Patients receiving APD | | 23 |
| Early death | | 1 |
| Evaluable patients | | 22 |
| Patients achieving normocalcaemia | | 18 |
| Effective APD dose | 5 mg | 1 |
| | 15 mg | 14 |
| | 2 × 15 mg | 3 |
| Patients resistant to APD | | 4 |

### Results

Figure 1 shows the fall in serum calcium during treatment. The median serum calcium before rehydration was 3.14 mmol$^{-1}$ (range 2.74–4.53). Intravenous rehydration led to only a minor improvement in serum calcium and 23 patients remained hypercalcaemic after rehydration with a median serum calcium of 3.05 mmol l$^{-1}$ (range 2.72–4.35) and received APD. The serum calcium increased during rehdyration in 8/25 (32%).

**Figure 1** Serum calcium response to APD (day 0) after rehydration (mean values ±s.e.m.).

Eighteen of 23 patients became normocalcaemic after APD with a steady fall in serum calcium over 48–96 h (Figure 1). Fourteen patients responded to a dose of 15 mg and 1 to 5 mg. In 7 patients additional doses of APD were necessary. Three of these patients achieved normocalcaemia after a second dose 15 mg (Table I). One patient died within 24 h of APD from septicaemia and rapidly progressive lymphangitis carcinomatosa. In 4 patients, hypercalcaemia appeared resistant to APD despite cumulative doses of 30, 80, 90 and 120 mg of APD. Three of these non-responders were also refractory to mithramycin, 2 of them dying from persistent hypercalcaemia. Temporary control of hypercalcaemia in 2 of the non-responders was achieved with cytotoxic chemotherapy. In 3 of these non-responders there was evidence of a predominantly humoral cause for hypercalcaemia; there was minimal radiological evidence of osteolytic bone disease and both tubular reabsorption of calcium and urinary phosphate loss were increased.

Urinary calcium excretion, shown as a molar ratio of calcium to creatinine in Table II and as $Ca_E$ in Figure 2, rose during rehydration but then fell towards normal as bone resorption was inhibited by APD. In 2 patients the calcium excretion remained high (molar ratio >0.5) indicating incomplete inhibition of bone resorption. One of these patients remained hypercalcaemic despite a total cumulative dose of 120 mg. APD and the other became hypercalcaemic again 7 days after a single dose of 5 mg.

Unexpectedly, hydroxyproline excretion remained elevated throughout the study (Figure 3). Figure 4 shows how $Ca_E$ and serum calcium related to each other during treatment in comparison to the normal range for these indices. Before rehydration this lay to the right of the normal range indicative of increased renal tubular reabsorption of calcium. Rehydration resulted in a fall in renal tubular reabsorption and restoration of the relationship to normal which was maintained while serum calcium fell.

**Table II** Biochemical assessment before and after rehydration, and after APD (mean values ± s.e.m.)

| *Study day* | *−2* | *0*[a] | *2* | *4* | *6* | *10* | *14* |
|---|---|---|---|---|---|---|---|
| *Serum* | | | | | | | |
| Calcium [mmol l$^{-1}$] | 3.28 (0.10) | 3.15 (0.09) | 2.88 (0.09) | 2.61 (0.08) | 2.51 (0.07) | 2.60 (0.09) | 2.74 (0.08) |
| Creatinine [μmol l$^{-1}$] | 108 (14) | 97 (16) | 84 (13) | 76 (10) | 62 (6) | 64 (8) | 63 (8) |
| Phosphate [mmol l$^{-1}$] | 0.94 (0.06) | 0.89 (0.07) | 0.80 (0.07) | 0.70 (0.04) | 0.70 (0.05) | 0.75 (0.09) | 0.83 (0.12) |
| Magnesium [mmol l$^{-1}$] | 0.69 (0.03) | 0.61 (0.04) | 0.59 (0.03) | 0.61 (0.04) | 0.58 (0.04) | 0.59 (0.10) | — |
| *Urine* | | | | | | | |
| $TmPO_4$/GFR[b] [mmol l$^{-1}$] | 0.59 (0.06) | 0.63 (0.05) | 0.65 (0.06) | 0.65 (0.07) | 0.59 (0.08) | 0.72 (0.11) | 0.74 (0.10) |
| Calcium/creatinine ratio [mmol mol$^{-1}$] | 1.06 (0.11) | 2.11 (0.20) | 1.41 (0.21) | 0.43 (0.17) | 0.68 (0.18) | 0.71 (0.14) | 1.17 (0.11) |
| Sodium excretion[c] [mmol l$^{-1}$] | 2.02 (0.69) | 3.70 (0.55) | 3.21 (0.52) | 2.15 (0.55) | 1.99 (0.42) | 0.94 (0.23) | 0.58 (0.22) |
| *Symptoms* | | | | | | | |
| Score[d] | 2.9 (0.3) | 2.0 (0.4) | 1.0 (0.3) | 0.7 (0.3) | 0.7 (0.3) | 0.9 (0.3) | 0.7 (0.3) |

[a]APD given on day 0; [b]$TmPO_4$/GFR = tubular maximum reabsorption of phosphate; [c]Sodium excretion = molar ratio of urinary sodium and creatinine × serum creatinine; [d]Symptom score = mean summation of WHO scores for nausea and vomiting, coma and confusion.

**Figure 2** Calcium excretion index ($Ca_E$) during rehydration and after APD (day 0). (mean values ±s.e.m.). $Ca_E$=molar ratio of urinary calcium to creatinine multiplied by serum creatinine.

**Figure 3** Urinary hydroxyproline excretion during treatment (mean values ±s.e.m.).

Table II summarises other biochemical data. Serum creatinine fell slightly during rehydration as the glomerular filtration rate (GFR) rose. The continued fall in serum creatinine after adequate rehydration suggests that the GFR did not return to normal until normocalcaemia was achieved. The serum phosphate fell during rehydration as the GFR rose, and was slow to return to normal as tubular function recovered and $TmPO_4$/GFR increased. Sodium excretion ($Na_E$) rose during rehydration as the salt deficit was replaced and gradually fell as the diuretic effect of excess calcium was removed and the i.v. saline reduced. Serum magnesium levels remained low throughout the study. Parathyroid hormone (PTH) was low or undetectable ($<400\,ng\,ml^{-1}$) in 22 patients. In 3 inconsistent, slight elevation of PTH of uncertain significance was noted.

All patients presented with symptoms attributable to hypercalcaemia. Subjective improvement invariably occurred after rehydration despite minimal improvement in serum calcium and continued until normocalcaemia was achieved (Table II). Many patients complained of persisting lethargy despite normocalcaemia. Although advanced malignancy may have been the cause of this it seems probable that persistent hypomagnasaemia contributed to this. In more than half of the patients serum magnesium levels remained below the normal range for 7 to 10 days after correction of serum calcium.

**Figure 4** Relation between calcium excretion ($Ca_E$, $\mu mol\,l^{-1}$ glomerular filtrate) and serum calcium. Points indicate mean values ±s.e.m. on day of study (APD given day 0). TmCa/GFR= tubular maximum reabsorption of calcium. Dotted line indicates normal range.

APD was tolerated without significant toxicity. A transient fever occurred in 2 patients. There was no gastro-intestinal toxicity, lymphopenia, renal impairment or local thrombophlebitis. The duration of action of APD was evaluable in 8 patients. Hypercalcaemia recurred 7–17 days after APD administration (median 11 days). In the other 10 patients achieving normocalcaemia additional systemic therapy, usually for rapidly progressing liver metastases, was necessary; hypercalcaemia recurred within 2 weeks in four.

### Discussion

Hypercalcaemia is an unpleasant and life-threatening complication of malignancy. Although the patient's prognosis may be poor, prompt effective treatment is necessary to relieve symptoms before appropriate anti-tumour therapy is started. The pathogenesis of hypercalcaemia is not fully understood (Mundy, 1985). The relative contributions of metastatic bone destruction (Ralston *et al.*, 1984), renal impairment, and humoral factor(s) remain contentious (Percival *et al.*, 1985*a*) and probably differ from patient to patient. The tumour type influences the predominant component and bone metastases are particularly common, although not invariable, in patients with hypercalcaemia secondary to breast cancer (Coleman & Rubens, 1987). The 4/25 (16%) incidence of hypercalcaemia without widespread bone metastases observed here is similar to that recorded in a recent retrospective review of hypercalcaemia (Coleman & Rubens, 1987).

Intravenous rehydration is the essential immediate treatment (Hosking *et al.*, 1982). Calcium has a powerful diuretic effect causing salt and water depletion. Urinary excretion of calcium is impaired by several factors – the fall in glomerular filtration, tubular damage secondary to the hypercalcaemia and sometimes the secretion by the tumour of a humoral factor with parathyroid hormone – like actions on the kidney (Mundy *et al.*, 1985).

Rehydration led to a small improvement in symptoms and renal function. The mean serum calcium fell by $0.13\,mmol\,l^{-1}$, less than in other studies (Hosking *et al.*,

1982; Percival *et al.*, 1984), with a rise in calcium in 8 patients. Immobilization may have contributed to the deterioration seen in these patients. Renal tubular reabsorption of calcium also fell following rehydration, resulting in a rise in urinary calcium excretion (Figure 2) and restoration of the normal relationship between serum and urinary calcium (Figure 4).

This study confirms the efficacy and lack of toxicity of APD with return of serum calcium to normal in 18/22 (82%) evaluable patients. A single 15 mg infusion of APD was effective in 15 patients. In these patients both serum and urinary calcium levels fell after 24 h, with a maximum effect by 4–5 days. In the other 3 patients who responded to APD an additional 15 mg was given after 48 h. Urinary calcium excretion had begun to fall in these patients and the second dose may not have been necessary; improvement in serum calcium followed over the next few days. The relatively slow onset of action was not of clinical importance in controlling hypercalcaemia in this study. A more rapid effect is possible if necessary by combining calcitonin and APD due to the direct effect of calcitonin on tubular function (Ralston *et al.*, 1986).

The four patients refractory to APD showed no significant improvement in serum calcium despite repeated doses. Three had a predominantly humoral mechanism responsible for hypercalcaemia with no radiological evidence of lytic bone disease, and five or fewer lesions on the bone scan. Urinary calcium excretion fell to normal in 3, suggesting adequate inhibition of bone resorption but the tubular effects of any humoral factor could not be expected to respond to APD. Relative resistance of humoral hypercalcaemia to bisphosphonates has been observed previously (Ralston *et al.*, 1985*a*). No adequate explanation can be given for failure of repeated doses (total 120 mg) to control hypercalcaemia in the remaining patient. This patient had extensive rapidly progressing lytic bone disease and a direct effect of breast cancer cells on bone resorption, late in the metastatic process, may have been responsible (Galasko & Bennett, 1976).

The persistence of increased hydroxyproline excretion is at variance with some previous studies (Sleeboom *et al.*, 1983; Ralston *et al.*, 1985*b*). Diet and extra-skeletal disease may have been significant additional sources of hydroxyproline. Despite control of hypercalcaemia, incomplete hydroxyproline response has been observed (Percival *et al.*, 1985*b*), and the value of hydroxyproline as an index of bone resorption in monitoring systemic therapy has been inconsistent (Coombes *et al.*, 1983).

Symptomatic response was rapid with both rehydration and correction of serum calcium by APD contributing. Hypomagnaesaemia results from tubular damage secondary to hypercalcaemia. Restoration of serum magnaesium to normal levels has been reported following APD (Sleeboom *et al.*, 1983) but did not occur in this study. Magnesium replacement may be worthwhile to minimise the lethargy induced by hypomagnaesaemia.

A reliable assay or label is not available to obtain pharmacokinetic data on the bisphosphonates. Previous reports have suggested a duration of action of several weeks but this has been after multiple administrations (Kanis *et al.*, 1986) or confused by confounding factors (Cantwell & Harris, 1987) including incomplete rehydration, concomitant steroids and different tumour types. The duration of action cannot be stated with certainty from this study, but in a small number of patients not requiring additional systemic therapy hypercalcaemia recurred after 10–14 days. The effects of larger doses of APD or prolonged (24 h) infusions were not addressed in this study but may differ from the 2 h infusion of 15 mg used here. The duration of control of osteolysis may be influenced by both the dose (Thiebaud & Jaeger, 1987) and infusion schedule of APD (Data on file, Ciba-Geigy Ltd., Horsham).

APD is a useful, safe drug in the management of acute tumour-induced hypercalcaemia. Control of recurrent hypercalcaemia with doses repeated fortnightly has been possible in 3 patients (unpublished observations) although eventual resistance developed after 2–3 months. The reversal and long-term control of osteolysis with bisphosphonates to decrease the morbidity from metastatic breast cancer is now a definite possibility. Based on the data reported here we are now testing a two weekly schedule of intravenous APD for this purpose.

We wish to thank Mr G. Chik and Ms S. Hoare for their technical assistance, the nursing staff for help with sample collection, and Ciba-Geigy Ltd. for supplies of APD.

### References

BIJVOET, O.L.M. (1980) Indices for the measurement of the renal handling of phosphate. In *Renal Handling of Phosphate*, Massry, S.G. & Freiseh, H. (eds) p. 1. Plenum: New York.

BODY, J.J. (1984). Cancer hypercalcaemia: Recent advances in understanding and treatment. *Eur. J. Cancer*, **20**, 865.

CANTWELL, B.M. & HARRIS, A.L. (1987). Effect of single high dose infusions of amino-hydroxypropylidene diphosphonate on hypercalcaemia caused by cancer. *Br. Med. J.*, **294**, 467.

CLEARY, J. & SAUNDERS, R.A. (1974). A simplified procedure for the measurement of total hydroxyproline in urine. *Clin. Chim Acta.*, **57**, 217.

COLEMAN, R.E. & RUBENS, R.D. (1987). The clinical course of bone metastases. *Br. J. Cancer*, **55**, 61.

COOMBES, R.C., DADY, P., PARSONS, C. & 4 others (1983). Assessment of response of bone metastases to systemic treatment in patients with breast cancer. *Cancer*, **52**, 610.

COOMBES, R.C., WARD, M.K., GREENBERG, P.B. & 4 others (1978). Calcium metabolism in cancer. Studies using calcium isotopes and immunoassays for parathyroid hormone and calcitonin. *Cancer*, **38**, 2111.

ELOMOAA, I., BLOMQVIST, C., GROHN, P. & 5 others (1983). Long-term controlled trial with diphosphonate in patients with osteolytic bone metastases. *Lancet*, **i**, 146.

ELTE, J.W.F., BIJVOET, O.L.M., CLETON, F.J., VAN OOSTEROM, A.T. & SLEEBOOM, H.P. (1986). Osteolytic bone metastases in breast carcinoma. Pathogenesis, morbidity and bisphosphonate treatment. *Eur. J. Cancer*, **22**, 493.

FISKEN, R.A., HEATH, D.A. & BOLD, A.M. (1980). Hypercalcaemia – A hospital survey. *Quart. J. Med.*, **49**, 405.

GALASKO, C.S.B. & BENNETT, A. (1976). Mechanisms of bone destruction in the development of skeletal metastases. *Nature*, **263**, 507.

GRANT, C.S., HOARE, S.A., MILLIS, R.R., HAYWARD, J.L. & WANG, D.Y. (1984). Urinary hydroxyproline and prognosis in human breast cancer. *Br. J. surg.*, **71**, 105.

HOSKING, D.J., COWLEY, A. & BUCKNALL, C.A. (1982). Rehydration in the treatment of severe hypercalcaemia. *Quart. J. Med.*, **200**, 473.

JUNG, A. (1982). Comparison of two parenteral diphosphonates in hypercalcaemia of malignancy. *Am. J. Med.*, **72**, 221.

KANIS, J.A., PERCIVAL, R.C., YATES, A.J.P., URWIN, G.H. & HAMDY, N.A.T. (1986). Effects of diphosphonates in hypercalcaemia due to neoplasia. *Lancet*, **i**, 615.

LEGHA, S.S., COWELL, K., BUGDAR, A.V. & BLUMERSCHEIN, G.R. (1981). Tamoxifen induced hypercalcaemia in breast cancer. *Cancer*, **47**, 2803.

MUNDY, G.R. (1985). Pathogenesis of hypercalcaemia of malignancy. *Clin. Endocrinol.*, **23**, 705.

MUNDY, G.R., IBBOTSON, K.J. & D'SOUZA, S.M. (1985). Tumour products and the hypercalcaemia of malignancy. *J. Clin. Invest.*, **76**, 391.

ORRELL, D.H. (1971). Albumin as an aid to the interpretation of serum calcium. *Clin. Chim. Acta.*, **35**, 483.

PEACOCK, M., ROBERTSON, W.D., NORDIN, B.E.C. (1969). Relation between serum and urinary calcium with particular reference to parathyroid activities. *Lancet*, **i**, 384.

PERCIVAL, R.C., YATES, A.J.P., GRAY, R.E.S. & 4 others (1985). Mechanisms of malignant hypercalcaemia in carcinoma of the breast. *Br. Med. J.*, **291**, 776.

PERCIVAL, R.C., PATERSON, A.D., YATES, Z.Z. & 5 others (1985). Treatment of malignant hypercalcaemia with clodronate. *Br. J. Cancer*, **51**, 665.

PERCIVAL, R.C., YATES, A.J.P., GRAY, R.E.S., NEAL, F.E., FOREST, A.R. & KANIS, J.R. (1984). Role of glucocorticoids in the management of hypercalcaemia. *Br. Med. J.*, **289**, 287.

RALSTON, S.H., ALZAID, A.A., GARDNER, M.D., BOYLE, I.T. (1986). Treatment of cancer associated hypercalcaemia with combined aminohydroxypropylidene diphosphonate and calcitonin. *Br. Med. J.*, **292**, 1549.

RALSTON, S.H., FOGELMAN, I., GARDNER, M.D. & BOYLE, I.T. (1984). Relative contribution of humoral metastatic factors to the pathogenesis of hypercalcaemia in malignancy. *Br. Med. J.*, **288**, 1405.

RALSTON, S.H., GARDNER, M.D. & BOYLE, I.T. (1985). Raised renal tubular calcium reabsorption predicts poor response to osteoclast inhibitors in cancer associated hypercalcaemia. *Scot. Med. J.*, **30**, 126.

RALSTON, S.H., GARDNER, M.D., DRYBURGH, F.J., JENKINS, A.S., COWAN, R.A. & BOYLE, I.T. (1985). Comparison of aminohydroxypropylidene diphosphonate, with mithramycin and corticosteroids/calcitonin in treatment of cancer-associated hypercalcaemia. *Lancet*, **ii**, 907.

REITSMA, P.H., BIJVOET, O.L.M., VERLINDEN-OOMS, S.H. & VAN DER WEE-PALS, L.J.A. (1980). Kinetic studies of bone and mineral metabolism during treatment with (3-amino-1-hydroxypropylidene)-1,1-bisphosphonate [APD] in rats. *Calcif. Tiss. Int.*, **32**, 145.

SLEEBOOM, H.P., BIJVOET, O.L.M., VAN OOSTEROM, A.T., GLEED, J.H. & O'RIORDAN, J.L.H. (1983). Comparison of intravenous (3-amino-hydroxypropylidene)-1,1-bisphosphonate and volume repletion in tumour-induced hypercalcaemia. *Lancet*, **ii**, 239.

THIEBAUD, D., JAEGER, PH., JACQUET, A.F. & BURCKHARDT, P. (1987). A dose response study with a single-day AHPrBP (APD) treatment in hypercalcaemia of malignancy. *Proc. 18th congress Union Therapeutics*, Geneva, 1987. Abstract C97.

WHO (1979). Offset publication. No. 48. *WHO Handbook for reporting results of cancer treatment*. WHO, Gevena.